# Supplementary material for: Chromothripsis during telomere crisis is independent of NHEJ, and consistent with a replicative origin
Source: Genome Res. 2019 May;29(5):737–49. doi: 10.1101/gr.240705.118 (PMC6499312; doi:10.1101/gr.240705.118)
Supplement: Supplemental Material [file supp_gr.240705.118_Supplemental_file_1.zip › contigs/annotated_contigs/DB96/contig.2.DB96_length_615_mean_cov_8.4487804878.docx]

**DB96_length_615_mean_cov_8.4487804878**

```
GCATCTCCCAGCCAAGGAACGCCAAGGAGGCCGGCAGCCGCCAGGAGCTGGAACCGGCAAGGAAGCAGCCTCCCCCAGAGCCTTCTAGA
 >chr12:124927497-124927806 + E=3e-174
GAATGTGGTCCTGCAGCACCTTGATTTCCAACTGCTGGCCTCCAGTACTGTGAGAGAAGAAAGTTCCTGCCATTTTCAGCTGCCCGGTT

CACGGCCGAGTGTTAAGGCAGCGCTGAGAAGCTAGTCCGGCGTGTGATCTTCCACAAACCGCGGTGCTGTCACTGCAGCTGGAAAACGA

CTCGGTGAGCATGAGGCCTCGGGGTGGTGGTGATGTGCAT|GC|TCCCCCTCCCTCTCTCCCCATCCGCGGCGGTGGCGGCGTCAGCAG
                                         >chr12:124873968-124874273 + E=8e-163
TGGTAAGAACAGATGAGGGTTCCAGAAAAACAGAGCTTCTCCACCCCCGGCAGACAGGGAGCAAACAGCTTCTTCCGCCATCATCCCCC

GCCGCCCGTCCCCCCACCCTCCGTGTCAGTTCCACCTGAGCTCTGCCCCCTGCCTGGCCTGATGCGGCCGAGGCTGACCGAGCCCTCTG

TCGGGGACTGTGGGTGGGCAGGGCTAGCCTCGCAATCCGGAGGGATCCCGGGATTTGAGGAGCCTTTGCACGGTGCACAT|CAG
```
